# The development of hepatic stellate cells in normal and abnormal human fetuses – an immunohistochemical study

**DOI:** 10.14814/phy2.12504

**Published:** 2015-08-11

**Authors:** Christine K C Loo, Tamara N Pereira, Katarzyna N Pozniak, Mette Ramsing, Ida Vogel, Grant A Ramm

**Affiliations:** 1 Department of Anatomical Pathology, Prince of Wales HospitalRandwick, Sydney, NSW, Australia; 2 Hepatic Fibrosis Group, QIMR Berghofer Medical Research InstituteBrisbane, Queensland, Australia; 3 Discipline of Pathology, School of Medicine, University of Western SydneySydney, NSW, Australia; 4 Department of Pathology, Aarhus University HospitalAarhus, Denmark; 5 Department of Clinical Genetics, Aarhus University HospitalAarhus, Denmark; 6 Faculty of Medicine and Biomedical Sciences, The University of QueenslandBrisbane, Queensland, Australia

**Keywords:** Circulating stem cells, cRBP-1, diaphragm, GFAP, Mesothelium

## Abstract

The precise embryological origin and development of hepatic stellate cells is not established. Animal studies and observations on human fetuses suggest that they derive from posterior mesodermal cells that migrate via the septum transversum and developing diaphragm to form submesothelial cells beneath the liver capsule, which give rise to mesenchymal cells including hepatic stellate cells. However, it is unclear if these are similar to hepatic stellate cells in adults or if this is the only source of stellate cells. We have studied hepatic stellate cells by immunohistochemistry, in developing human liver from autopsies of fetuses with and without malformations and growth restriction, using cellular Retinol Binding Protein-1 (cRBP-1), Glial Fibrillary Acidic Protein (GFAP), and *α*-Smooth Muscle Actin (*α*SMA) antibodies, to identify factors that influence their development. We found that hepatic stellate cells expressing cRBP-1 are present from the end of the first trimester of gestation and reduce in density throughout gestation. They appear abnormally formed and variably reduced in number in fetuses with abnormal mesothelial Wilms Tumor 1 (WT1) function, diaphragmatic hernia and in ectopic liver nodules without mesothelium. Stellate cells showed similarities to intravascular cells and their presence in a fetus with diaphragm agenesis suggests they may be derived from circulating stem cells. Our observations suggest circulating stem cells as well as mesothelium can give rise to hepatic stellate cells, and that they require normal mesothelial function for their development.

## Introduction

Liver stromal cells influence many biological and pathological processes (Wang et al. 2010; Kiyohashi et al. 2013). The embryological origin and development of mesenchymal cells of the liver is not fully established (Yin et al. 2013); this includes the cells responsible for liver wound healing, hepatic stellate cells (Friedman 2008). Animal experiments have shown that mesodermal cells delaminate from the mesothelium to form hepatic stellate cells and perivascular mesenchymal cells (Asahina et al. 2009, 2011). This process was also illustrated in *wt1*^−/−^ mice (Ijpenberg et al. 2007) and in human fetuses (Loo and Wu 2008). We previously reported WT1 abnormalities in liver mesothelium of human fetuses with bilateral renal agenesis and cardiac defects (Loo et al. 2012b). We also recently reported a 27 week gestation fetus with bilateral diaphragm hernia probably due to failure of development of the pleuroperitoneal folds (Loo et al. 2015), similar to *wt1*^−/−^ mice. In the current study, we describe the development of hepatic stellate cells in these fetuses in more detail and in a much larger cohort of fetuses, including control fetuses and fetuses with other anomalies, using immunohistochemistry for hepatic stellate cell markers, cellular retinol-binding protein-1 (cRBP-1), glial fibrillary acidic protein (GFAP) and alpha-smooth muscle actin (*α*SMA). It has been suggested that stellate cells derive from bone marrow (Baba et al. 2004; Miyata et al. 2008) and show similar features to marrow stromal cells which support haemopoiesis (Kordes et al. 2013). Using Mesoderm Posterior 1 Homolog (MesP1) as a marker, it has been shown that stellate cell precursors originate from posterior mesoderm and reach the liver via the septum transversum and diaphragm in mice (Asahina et al. 2009), probably via the body wall and pleuroperitoneal membranes (Ijpenberg et al. 2007). During the first trimester, mesenchymal stem cells (MSC) from the aorta-gonad-mesonephros region (AGM) migrate to the liver to establish a stromal network for hemopoietic cells (Oostendorp et al. 2002; Durand et al. 2006), as the liver is the main site of haemopoiesis in midgestation (Marshall and Thrasher 2001). It is known that MesP1 expressing progenitor cells contribute to hemopoietic stromal cells in the yolk sac and AGM in mice (Chan et al. 2013). We have studied hepatic stellate cells in normal and abnormal human fetuses to identify factors that influence their development. We found intravascular cells morphologically similar to hepatic stellate cells in normal and abnormal fetuses, including a fetus with diaphragm agenesis due to failure of development of the pleuroperitoneal folds (as reported Loo et al. 2015). We discuss the hypothesis that hepatic stellate cells may be derived from circulating MSC.

## Patients and Methods

The archived records of the Department of Anatomical Pathology, Royal Brisbane and Women’s Hospital (RBWH), Queensland, Australia were analyzed for fetal autopsy cases of nonmacerated fetuses. Fetuses with intra-uterine growth restriction, fetuses from miscarriages without malformations (controls) and fetuses with various other anomalies were identified over a 2-year period (2009–2010). Also included are renal agenesis and control cases that we have previously reported (Loo et al. 2012b) and a bilateral diaphragm agenesis fetus we recently described (Loo et al. 2015). Similarly, autopsy files of Anatomical Pathology, Prince of Wales Hospital (PoWH) in NSW, Australia were analyzed over a 9-year period (2005–2013) for autopsies of nonmacerated fetuses where parents had given consent for research use of tissues. All fetal autopsies in these hospitals are performed or supervised by specialist perinatal/pediatric pathologists and karyotype or subtelomere multiplex ligation-dependent probe amplification (MLPA) screen and babygram Xrays are routinely obtained. Additional cases were sent from Aarhus University Hospital – 3 controls, 4 fetuses with short rib polydactyly syndrome including 1 with ductal plate anomaly. In total, this study included 43 control fetuses and 59 fetuses with abnormalities, which included 10 with bilateral renal agenesis, 7 with combined bilateral renal agenesis and heart defect, 8 with ductal plate malformation, 5 with growth restriction, 6 with diaphragm hernia, and 23 with other anomalies, including brain malformations, heart malformations, VATER/VACTERL (acronym for Vertebral anomalies, Anal atresia, Tracheo-Esophageal fistula and Renal/radial anomalies/Vertebral, Anal, Cardiac, Tracheo-Esophageal, Radial/renal and Limb anomalies) association, chromosomal disorders, skeletal malformations, and metabolic syndromes.

### Immunohistochemistry

Immunoperoxidase stains were performed as previously described (Ramm et al. 2009). In preliminary studies, we found technical difficulties, lack of specificity or expression limited to certain periods of development with other potential markers of hepatic stellate cells (results not shown); including NCAM (neural cell adhesion molecule), desmin, synaptophysin, and CD133 (cluster of differentiation 133) (Geerts 2001; Kordes et al. 2007; Zhao and Burt 2007), D2-40 (D2-40 antibody), CD34 (cluster of differentiation 34), S-100 protein (Soluble in saturated (100%) ammonium sulfate solution), caldesmon, and myogenin. We chose cRBP-1 (to mark quiescent stellate cells), GFAP and *α*SMA (to mark activated stellate cells) as these are recognized markers of human hepatic stellate cells that were consistent and reliably expressed in paraffin-embedded fetal and adult tissue in our laboratory.

Paraffin-embedded tissue sections were cut at 3 *μ*m and stained with antibodies to cRBP-1 (Santa Cruz [FL-135] rabbit antihuman polyclonal sc-30106, 1:50 dilution at room temperature for 1 h) and *α*SMA (Dako, clone 1A4, dilution 1:100 no retrieval) by routine methods. The number of hepatic stellate cells expressing cRBP-1 antigen was manually counted in 10 contiguous hpf (high power field) (×40 objective) by one of the investigators (CL) – hepatic stellate cells were only counted if the nuclei and processes were present. In younger fetuses, limited tissue was available for analysis so only 10 hpf were initially assessed in each fetus. As reduced numbers of hepatic stellate cell numbers were observed in some fetuses with bilateral renal agenesis and diaphragm agenesis, these fetuses were therefore analyzed separately with 50 hpf counted for each fetus and matched controls.

*α*SMA expression in lobular cells was scored as previously described (Loo et al. 2012b). Briefly, *α*SMA expression was scored as: occasional (score 0.5 - rare perisinusoidal cells express this antigen, weak (1+, *α*SMA is positive mainly around portal tracts and central veins with scattered cells positive in other parts of the lobule), moderate (2+, *α*SMA is positive in some perisinusoidal cells distant from portal tracts and central veins) and strong (3+, *α*SMA is expressed by most perisinusoidal cells).

GFAP immunoperoxidase stains were performed on 4 *μ*m paraffin sections dewaxed in xylene and rehydrated through descending concentrations of alcohol solutions to water. Sections were heat retrieved for 15 min at 105°C in citrate buffer pH6.0 using a Biocare Medical Decloaking Chamber. Endogenous peroxidase was quenched using a 1% hydrogen peroxide solution and nonspecific binding blocked with normal donkey serum. The primary antibody, Biocare Medical mouse anti-GFAP diluted 1:150 was applied for 1 h then detected using Vector Impress Mouse HRP Polymer secondary. Signals were visualized with DAB (Diaminobenzidine) and the sections lightly counterstained with hematoxylin before dehydrating and coverslipping. The prevalence (absent, scant or normal) of cells with hepatic stellate cell morphology or progenitor characteristics (large round nuclei, intrasinusoidal or intravascular location, little cytoplasm) were assessed.

### Dual immunofluorescence for *α*SMA/GFAP and *α*SMA/cRBP-1

Dual *α*SMA/GFAP and *α*SMA/cRBP-1 immunofluorescence (IF) was performed in six cases including one control, one renal agenesis fetus without cardiac anomaly or WT1 defect, one renal agenesis fetus with cardiac anomaly with retained WT1 mesothelial expression, two renal agenesis fetuses with WT1 and cardiac defects and one fetus with right-sided congenital diaphragm hernia.

Liver sections were deparaffinized in xylol, rehydrated by alcohol gradient, subject to peroxidase blocking (Biocare Medical) and Background Sniper (Biocare Medical) with 2% BSA. Sections were incubated with mouse anti-*α*SMA primary antibody (Biocare Medical), followed by a secondary MACH antimouse HRP (Biocare Medical) antibody and TSA™-FITC signal amplification (Life Technologies).

After microwave treatment with citrate pH 6.0 two separate protocols were carried out using rabbit antihuman cRBP-1 (Santa Cruz) or mouse anti-GFAP (Biocare Medical) followed by a secondary MACH anti-rabbit HRP (Biocare Medical) antibody and TSA™-Cy3 signal amplification (Life Technologies). After a second microwave treatment slides were stained with DAPI. Sections were examined using confocal microscope Zeiss 780NLO and captured using Aperio FL slide scanner (Aperio Technologies).

Signed informed consent was obtained from the parents for each case. This project was approved by the human research ethics committees of RBWH, PoWH and QIMR Berghofer Medical Research Institute. Parental consent was obtained for the fetuses from Aarhus University Hospital.

## Results

### Control fetuses

**Figure 1:**
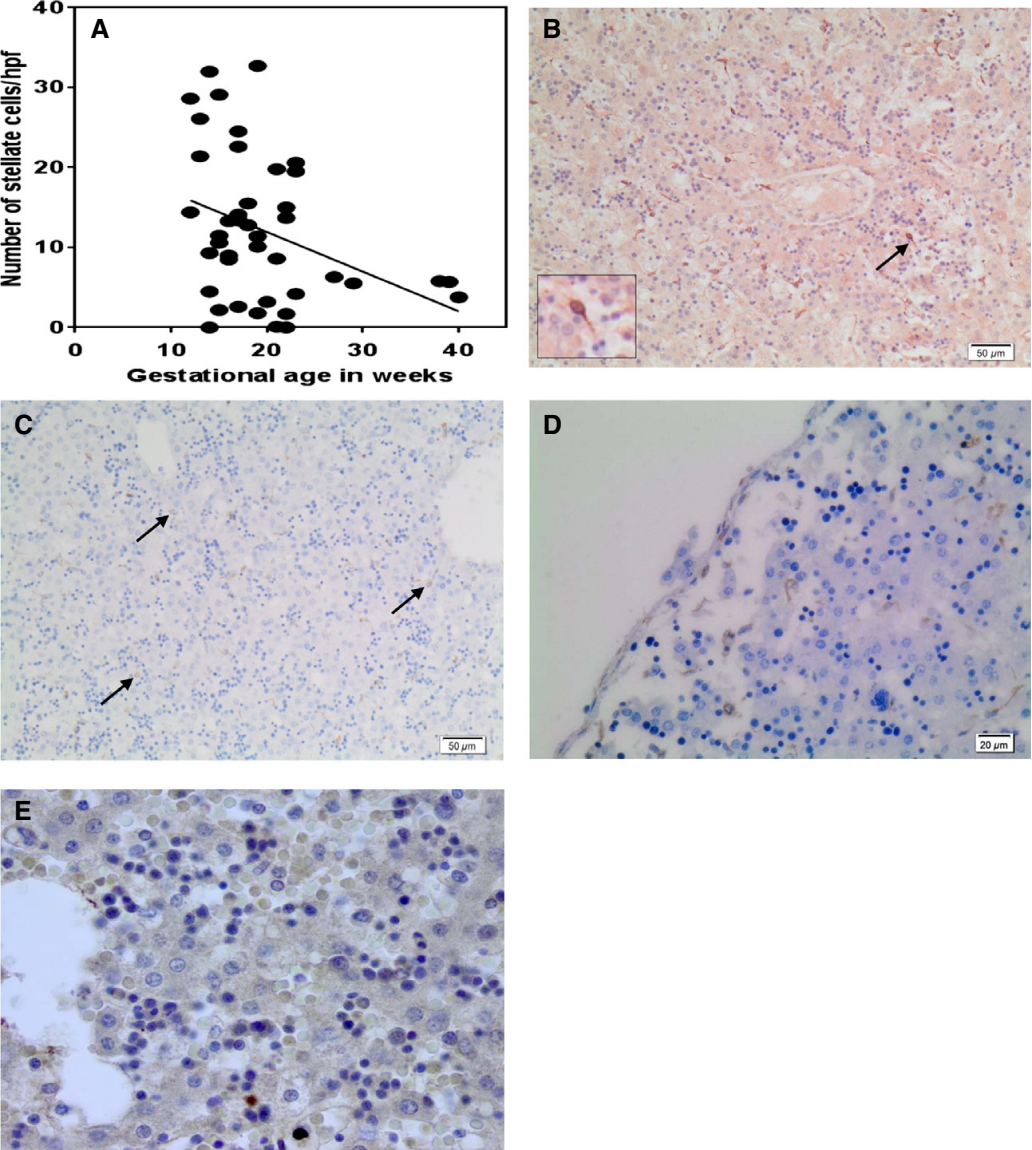
Hepatic Mesenchymal Cells in Control Fetuses. (A) cRBP-1-positive cell numbers at different gestational ages in control fetuses. The mean number of stellate cells/hpf from 10 hpf was calculated for each control fetus. Although there is wide variation in numbers of stellate cells at each gestational age, there is a statistically significant reduction in stellate cell density with increasing gestational age (*r* = −0.3576, *P* = 0.0186). Hepatic mesenchymal cells stained for cRBP-1 (B), GFAP (C, D), and *α*SMA (E) in a 19 week gestational control fetus. (B) Numerous hepatic stellate cells are present, showing oval nuclei and long cell processes expressing cRBP-1 antigen (arrow and inset). (C) Numerous stellate-shaped perisinusoidal cells expressing GFAP. Rounded GFAP+ve cells in the sinusoidal spaces (arrows) show similar nuclear features and appear to be transitional forms between intravascular and perisinusoidal cells. (D) GFAP stain showing some submesothelial cells expressing this antigen. There are relatively abundant stellate cells beneath the liver capsule. (E) *α*SMA shows scant stellate cells in the liver lobules. (score 0.5 for *α*SMA); Original magnification ×400.

In control fetuses, the number of quiescent hepatic stellate cells (expressing cRBP-1) decreased with gestational age (Fig.1A). Although there was wide variation between fetuses especially in the earlier gestational ages this trend was statistically significant (*r* = −0.3576, *P* = 0.0186). Abundant hepatic stellate cells with long cell processes were seen in the perisinusoidal space with cRBP-1 immunoperoxidase stain (Fig.1B). The distribution of hepatic stellate cells varied considerably, but they appeared to be concentrated in the subcapsular areas in early gestation (not shown). GFAP mostly corroborated cRBP-1 findings in control fetuses (Table1). GFAP antigen was also expressed in intravascular cells within sinusoids in most cases (Fig.1C). These were sometimes difficult to distinguish from hepatic stellate cells showing a morphological continuum from intravascular cells with large rounded nuclei to stellate-shaped perisinusoidal cells (Fig.1C). Stellate cells also appeared more abundant beneath the capsule in GFAP stained sections, especially in younger fetuses (Fig.1D). *α*SMA results have previously been reported in these controls (Loo et al. 2012b). Perivascular/perisinusoidal cells expressing *α*SMA were mostly found around the portal tracts and central veins (Fig.1E) at all gestational ages, with only scattered cells found in other parts of the lobules in some fetuses, in keeping with other reports (Villeneuve et al. 2009), but they also appeared concentrated in the subcapsular area in younger fetuses (not shown).

**Table 1:** Clinical details and expression of cRBP-1, GFAP, *α*-SMA, and WT1 in Renal Agenesis Cases versus Controls (modified from Loo et al. 2012b)

Cases	Gestational age	Clinical details	cRBP-1	GFAP	SMA	Wt1 expression in mesothelium
		Fetuses with Bilateral renal agenesis and cardiac defects	Mean +/− SEM HSC/hpf			
(1)	17	Small bladder, small uterus and gonads, single umbilical artery, 46,XX	0.7 ± 0.1	No stellate cells, possible scant progenitors	1+	Absent
		Cardiomegaly				
(2)	19	Pericardial effusion, CCAM type 2 (RUL, RML), shortened mesentery of intestines, small thymus, Enlarged liver and spleen, enlarged heart with tricuspid and mitral regurgitation, 46,XY	7.3 ± 0.6	Numerous well formed stellate cells and progenitors	3+	Reduced
(3)	19	IUGR, arrhinencephaly, cleft lip and palate, small tongue, large abnormal auricles, Holoprosencephaly,	0.7 ± 0.2	Many cells positive, possibly progenitors in sinusoids. Few poorly formed stellate cells	3+	Reduced
		postaxial polydactyly left hand, bilateral clinodactyly, abnormal fifth digit right hand, incomplete fissuring of lungs, truncus arteriosus, VSD. 46,XX				
(4)	19	Absent ureters, bladder, Partial syndactyly digits 2–5 on both feet, right lung malformation, right sided liver and shift of other abdominal organs to left, velamentous cord insertion, no skeletal malformations, Globular heart with thickened wall, RV hypoplasia and abnormal tricuspid valve, thickened fibrotic endocardium, 46, XY	1.0 ± 0.2	Abundant well formed stellate cells, possible progenitor cells in sinusoids	3+	Reduced
(5)	20	Compressed face, globoid head, low set ears, small jaw, nuchal oedema, stenosis of segment of small intestine, right ventricular fibroelastosis, mild pulmonary valve stenosis, cardiomegaly, muscular VSD. 46,XY	1.4 ± 0.2	Few stellate cells, numerous progenitor cells in sinusoids	3+	Positive
(6)	23	Slightly small for gestational age, pre-axial polydactyly of left foot, malrotation of gut, 10 ribs on right, 9 ribs on left, segmentation abnormalities lower thoracic and lumbosacral spine, hydrocephalus, single umbilical artery, pulmonary valve stenosis, overriding aorta, perimembranous VSD 46,XY	0	Occas cells within sinusoids express GFAP but very few stellate cells	3+	Absent
(7)	41	Twin, craniopagus, 38/40 at birth, surgery 23/7 after birth. Baby had bilateral renal agenesis, small bladder, mild malrotation of gut, cardiomegaly 46,XX	0.7 ± 0.1	No stellate cells	3+	Reduced
		Fetuses with bilateral renal agenesis without cardiac defects				
(8)	18	Absent uterus and fallopian tubes, both ovaries present but elongated 46,XX	8.9 ± 0.6	Numerous progenitors, and stellate cells some with long processes	2+	Positive
(9)	19	Small bladder, marginal cord insertion, 46,XY	12.2 ± 1.0	Numerous well formed stellate cells	1+	Positive
(10)	19	Limb anomalies, imperforate anus, single umbilical artery, absent gallbladder, 46,XY	2.0 ± 0.3	Small clusters of stellate cells and progenitors	1+	Positive
(11)	20	Absent ureters, small bladder, anal atresia, sacral agenesis, hemivertebrae 46,XY (?VATER)	14.0 ± 1.1	Abundant stellate cells some progenitor cells	2+	Positive
(12)	20	Small bladder, small prostate, normal testes 46,XY	2 ± 0.3	Numerous well formed stellate cells, few possible progenitors	2+	Positive
(13)	20	No other anomalies, 46,XY	7.68 ± 0.8	Numerous well formed stellate cells, few progenitors	2+	Positive
(14)	20	Absent bladder, small but normal thymus 46,XY add(9)(p24).ish add(9)(pter-,p16+)	13.34 ± 0.9	Numerous stellate cells and progenitors	2+	Positive
(15)	21	Small bladder, 46,XY	17.9 ± 0.9	Not done	2+	Positive
(16)	21	Absent bladder and ureters, 46,XY	5.6 ± 0.7	Not done	2+	No mesothelium in section
Control	Gestational age	Control fetuses with no malformations	Mean +/− SEM	GFAP	SMA	Wt1 expression in mesothelium
(17)	15	Acute chorioamnionitis	6.9 ± 0.7	Numerous well formed stellate cells	0	Positive
(18)	16	Female, miscarriage	12.2 ± 1.0	Few stellate cells or progenitors	1+	Positive
(19)	16	Female, congenital bronchopneumonia	9.1 ± 0.7	Numerous well formed stellate cells + progenitors	1+	Positive
(20)	17	Placental infarct	17.4 ± 1.0	Numerous well formed stellate cells, few progenitors	0	Positive
(21)	18	Male, twin delivered a week earlier	11.0 ± 0.7	Numerous well formed stellate cells few progenitors	1+	Positive
(22)	19	Female, maternal fibroids, placental infarcts	18.5 ± 1.1	Abundant stellate cells (some with processes) + progenitors	1+	Positive
(23)	19	Female, acute chorioamnionitis, miscarriage, PV bleed	6.0 ± 0.7	Numerous well formed stellate cells	1+	Positive
(24)	20	Cervical incompetence	2.1 ± 0.2	Numerous stellate cells, no cell processes	0	Positive
(25)	21	Male, acute chorioamnionitis	7.5 ± 0.7	Not done	0	Positive
(26)	23	Acute chorioamnionitis	12.6 ± 0.9	Numerous well formed stellate cells, some progenitors	1+	Positive
(27)	23	Male, acute chorioamnionitis	10.3 ± 0.8	Abundant well formed stellate cells	1+	Positive
(28)	29	Male, PET, abruption	5.5 ± 0.3	Not done	1+	Positive

?Vater, possible VATER case.

### Renal agenesis fetuses

**Figure 2:**
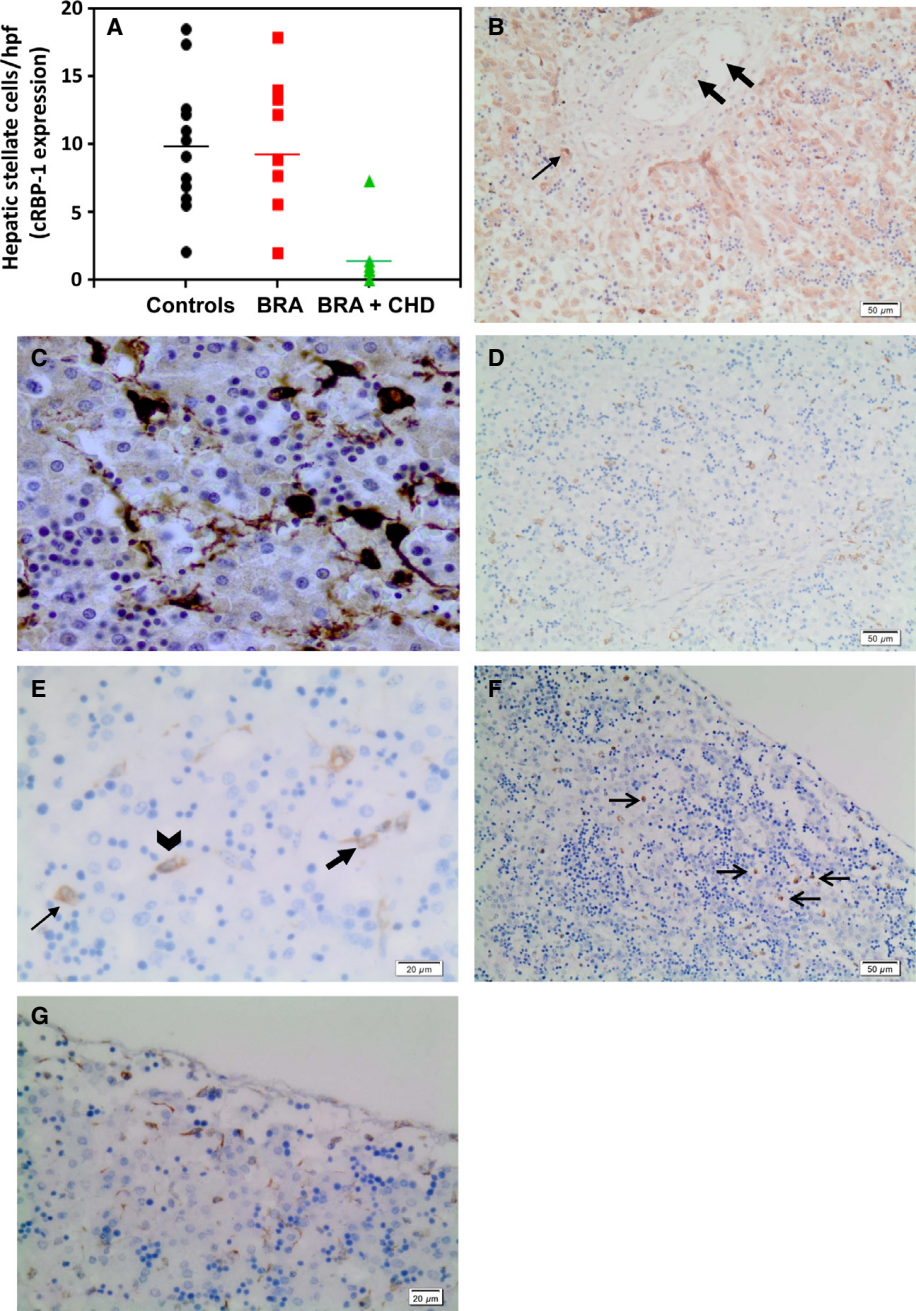
Hepatic Mesenchymal Cells in Renal Agenesis Fetuses. (A) cRBP-1-positive cell numbers at different gestational ages in Renal Agenesis Fetuses. Numbers of hepatic stellate cells/hpf expressing cRBP-1 in fetuses with bilateral renal agenesis, with or without cardiac defects compared to matched control fetal liver. Mean of 50 hpf is provided for each case. There were significantly fewer hepatic stellate cells in cases of bilateral renal agenesis fetuses with cardiac defects (BRA + CHD; gestational ages 17–41 weeks) versus both controls (gestational ages 15–29 weeks) and bilateral renal agenesis fetuses without cardiac defects (BRA; gestational ages 18–21 weeks) (ANOVA, *P *=* *0.0023), independent of gestational age. Results for individual fetuses are presented in Table1. Hepatic mesenchymal cells stained for cRBP-1 (B), *α*SMA (C) and GFAP (D–G) in renal agenesis fetuses. (B) cRBP-1 shows fewer stellate cells and these have shorter cell processes (small arrow) compared with control (see Fig.1B). Circulating mesenchymal cells are present in the blood vessel (large arrow). (C) There are many more perisinusoidal cells expressing *α*SMA (score 3 for *α*SMA) versus the control fetus (see Fig.1E); Original Magnification, ×400. (D) Many intravascular and perisinusoidal cells, some with stellate morphology, expressing GFAP. (E) High power view of (D) showing GFAP+ve intravascular cells (small arrow) and stellate shaped perisinusoidal cells with stellate morphology (large arrow) and cells of the transitional forms between intravascular and perisinusoidal cells (arrowhead). (F) In a fetus with bilateral renal agenesis, cardiac and WT1 defects, there are numerous round intravascular cells expressing GFAP antigen (arrows), while stellate cells in the perisinusoidal space with characteristic long processes are scant. These are not concentrated beneath the mesothelium as in control cases (as seen in Fig.1D). (G) GFAP immunohistochemistry shows abundant stellate cells beneath the capsule in a renal agenesis fetus without cardiac or WT1 defects.

The clinical features, WT1 and *α*SMA data for renal agenesis fetuses have been previously reported (Loo et al. 2012b) and are reiterated in Table1 along with data for cRBP-1 and GFAP for ease of comparison. The cRBP-1 immunoperoxidase stains showed marked reduction in hepatic stellate cell numbers and abnormal formation of stellate cells in fetuses with combined bilateral renal agenesis and congenital heart defects (with decreased WT1 expression in liver mesothelium in most of these cases), compared to control fetuses (*P* < 0.01) and other fetuses with bilateral renal agenesis without heart or WT1 defects (*P* < 0.01) (Fig.2A, Table1). The abnormally formed stellate cells (Fig.2B) had rounded cell bodies and lacked the long processes and oval cell bodies seen in Control cases (Fig.1B). There was also increased expression of *α*SMA in the liver lobules in renal agenesis fetuses as we previously described (Loo et al. 2012b). This is further illustrated in another photomicrograph (Fig.2C). Immunoperoxidase stains for GFAP showed abundant cells in the liver including possible progenitor cells within blood vessels, often more than seen using cRBP-1 as a marker (Fig.2D and E, Table1). However, in two fetuses where mesothelium was present, there were scant cells expressing GFAP in the subcapsular area (Fig.2F) compared to other fetuses without renal agenesis or renal agenesis fetuses without WT1 or cardiac defects (Fig.2G). GFAP antigen was not expressed in mesothelium.

**Figure 3:**
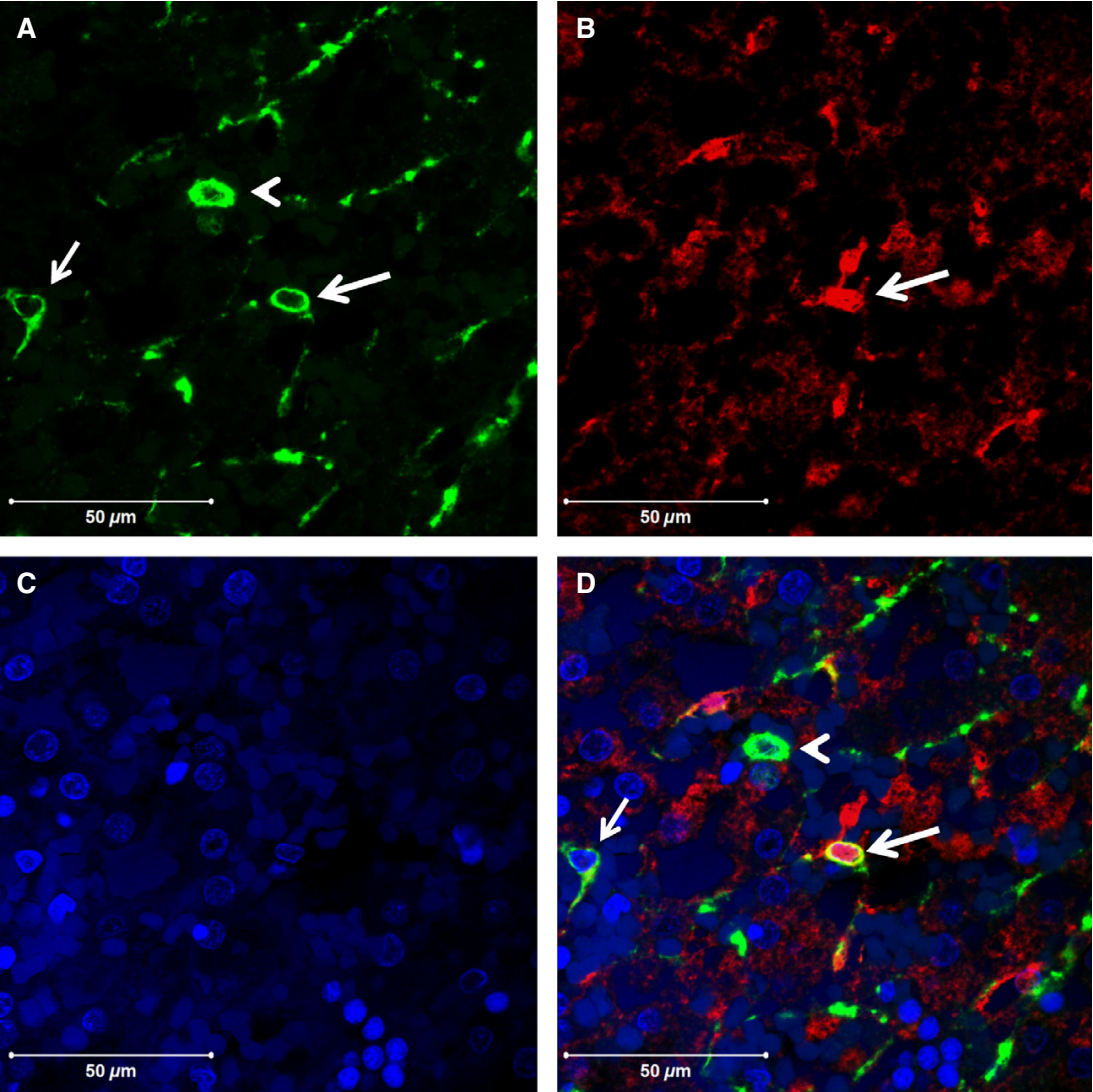
Colocalization of *α*SMA and cRBP-1. Dual immunofluorescence for (A) *α*SMA (green), (B) cRBP-1 (red) and (C) DAPI (blue) in renal agenesis fetus, showing a large round cell (mesenchymal stem cell, arrowhead) and an activated stellate cell (arrow) expressing *α*SMA (A). There is another similar round cell coexpressing both antigens (large arrow), presumably a stem cell in early transition to a stellate cell (D, merge). Stellate cells expressing cRBP-1 antigen alone are also seen (B). (Original magnification, ×630).

**Figure 4:**
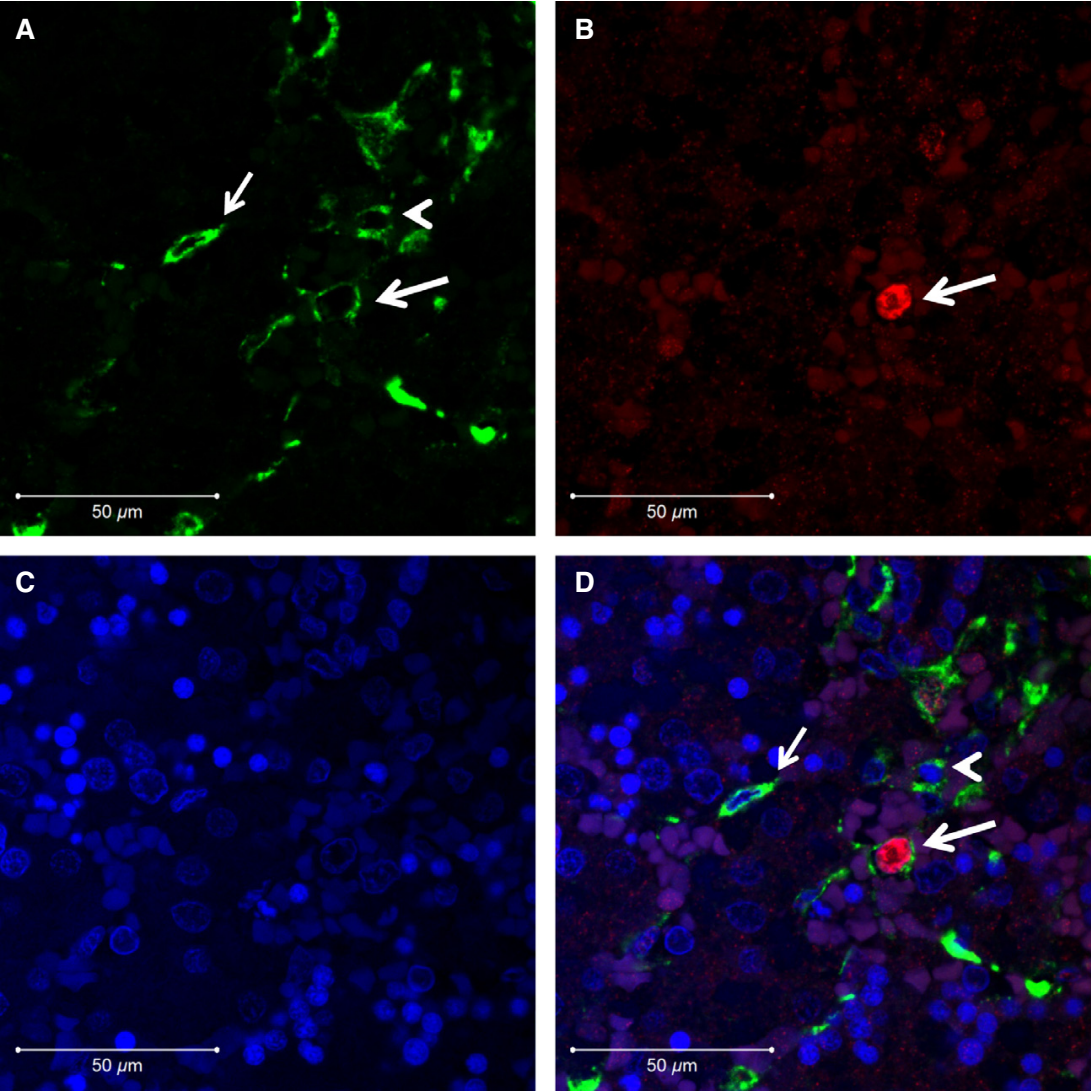
Colocalization of *α*SMA and GFAP. Dual immunofluorescence for (A) *α*SMA (green), (B) GFAP (red) and (C) DAPI (blue) in renal agenesis fetus showing a hepatic stellate cell (arrow) and mesenchymal stem cell (arrowhead) expressing *α*SMA only (A), and a mesenchymal stem cell coexpressing both *α*SMA and GFAP (B) possibly in transition to a hepatic stellate cell (large arrow) and (D, merge). (Original magnification, ×630).

Dual immunofluorescence showed co-expression of cRBP-1 and *α*SMA in scattered cells, which are presumably in various stages of transition between stellate cells and mesenchymal cells based on morphological and immunophenotypic features, in bilateral renal agenesis (Fig.3), whereas co-expression of these antigens was present in fewer hepatic stellate cells in control fetuses. There were also many cells co-expressing GFAP and *α*SMA including intravascular cells, some vascular smooth muscle cells and stellate cells (Fig.4).

### Fetuses with diaphragmatic hernia

**Figure 5:**
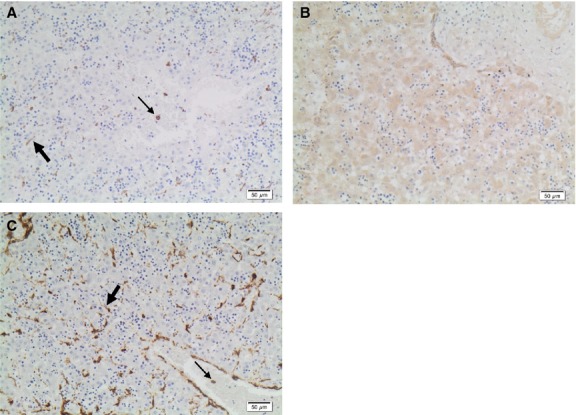
Hepatic mesenchymal cells in main liver of 27 week diaphragm agenesis fetus showing (A) GFAP, (B) cRBP-1, and (C) *α*SMA expressing mesenchymal cells. (A) GFAP is expressed in large, rounded intravascular cells (small arrow), and perisinusoidal stellate cells (large arrow). (B) cRBP-1 is weakly expressed in ductal plate cells but there are very scant perisinusoidal stellate cells expressing this antigen. (C) Increased numbers of stellate-shaped perisinusoidal cells express *α*SMA antigen (large arrow) as well as a few large, rounded intravascular cells, including one in a central vein in the lower right of the photomicrograph (small arrow).

**Figure 6:**
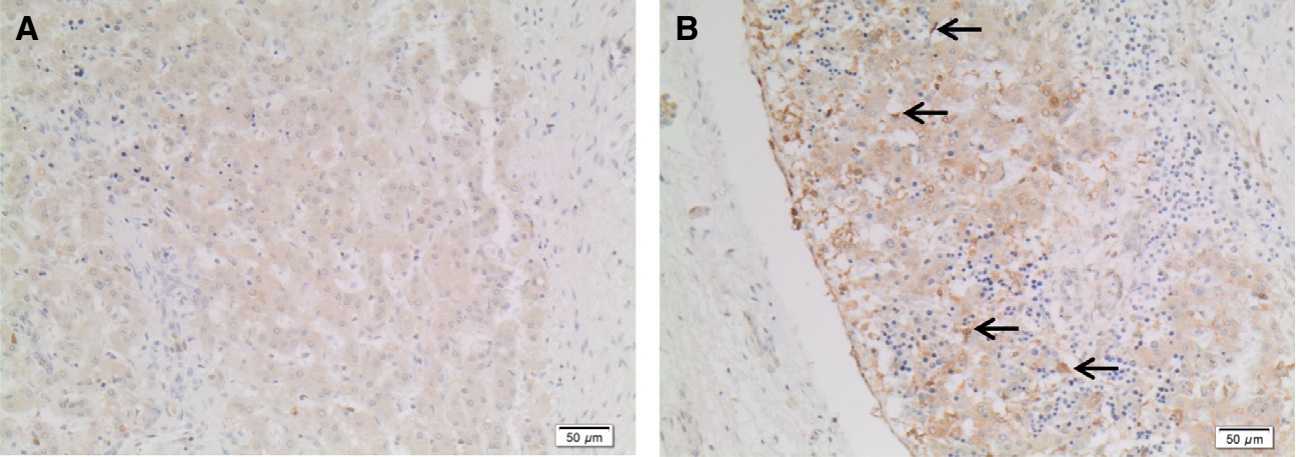
cRBP-1 immunoperoxidase stains of ectopic liver nodules in diaphragm agenesis fetus. (A) Scant stellate cells in a nodule without mesothelium. (B) Many stellate cells (arrows) and overlying mesothelium express cRBP-1 antigen.

We recently described a case study of a 27 week fetus with bilateral diaphragm agenesis (Loo et al. 2015). In this same fetus, we have now identified abundant hepatic stellate cells expressing GFAP (Fig.5A) in the liver, although we had previously found that normally differentiated stellate cells expressing cRBP-1 were scant (Loo et al. 2015) (Fig.5B). We had also previously described that cells expressing *α*SMA antigen in this same fetus were increased in liver lobules (Loo et al. 2015), further illustrated in Fig.5C. In this fetus, we also noted occasional large cells expressing GFAP and *α*SMA antigens within blood vessels (Fig.5A and C, respectively). This fetus showed ectopic liver nodules, some of which were covered by mesothelium (Loo et al. 2015). The cRBP-1 immunoperoxidase stain showed abundant quiescent stellate cells in ectopic liver covered by mesothelium but not in ectopic liver nodules without mesothelium (Loo et al. 2015), further illustrated in Figure6. In another fetus with right-sided diaphragm agenesis and displacement of liver into the thorax, many stellate cells were seen with GFAP but not cRBP-1 antibody, many showing features of progenitor cells or transitional forms between progenitor cells and stellate cells (results not shown) as seen in renal agenesis fetuses (as in Figs.3, 4). Stellate cell numbers (assessed by cRBP-1 immunoperoxidase stains) were slightly reduced in a fetus with a giant omphalocele that contained the displaced liver, but within the normal range in other fetuses with isolated, smaller diaphragm hernias.

### Fetuses with other anomalies

The number of hepatic stellate cells in fetuses with intrauterine growth restriction or ductal plate malformation was slightly increased compared to Controls (results not shown). The expression of *α*SMA was increased in a 14 week fetus with triploidy and varied in fetuses with ductal plate malformation (results not shown). We did not detect any significant differences in *α*SMA expression in fetuses with other anomalies. The number of stellate cells in fetuses with other anomalies was similar to those in Controls.

## Discussion

The embryological origin of hepatic stellate cells is difficult to investigate because there are no specific markers of fetal stellate cells. Unlike adult stellate cells, fetal stellate cells lack lipid droplets (Kato et al. 1985) and show variable antigen expression during development (Golbar et al. 2012). Hepatic stellate cells from fetal rat liver at E13–14 (embryonic day 13–14) do not express GFAP in vivo (Kubota et al. 2007) unlike adult rodent stellate cells (Zhao and Burt 2007; Yang et al. 2008) but GFAP expression can be induced in cultures from E11 mouse stellate cells (Suzuki et al. 2008). cRBP-1, claimed to be a relatively specific marker for quiescent human hepatic stellate cells in paraffin-embedded tissue sections (Van Rossen et al. 2009), is also expressed in portal and sinusoidal myofibroblasts in some circumstances (Uchio et al. 2002; Schmitt-Graff et al. 2003) and weakly in activated stellate cells (Van Rossen et al. 2009). *α*SMA is a marker of myofibroblastic cells including activated stellate cells, perivascular mesenchymal cells (Lepreux et al. 2004; Asahina et al. 2009) and smooth muscle cells. GFAP is a marker of human hepatic stellate cells, possibly in early activation phase (Carotti et al. 2008).

Our current studies in human fetuses suggest that stellate cells derive from mesothelium. Stellate cells were concentrated beneath the liver capsule in early gestation and expressed similar antigens to submesothelial and mesothelial cells. Our previous study of the fetus with bilateral diaphragm agenesis also supported this suggestion, as this fetus also had ectopic liver nodules showing abundant hepatic stellate cells expressing cRBP-1 antigen in the ectopic liver nodules covered by mesothelium but scant and abnormally formed stellate cells in liver nodules without mesothelium using cRBP-1 antigen (Loo et al. 2015), further illustrated in Figure6.

Liver mesenchymal cells were abnormally formed in renal agenesis fetuses with reduced WT1 liver mesothelial expression, generally lacking stellate cells that express cRBP-1. These fetuses also showed increased expression of *α*SMA in the lobules as previously described (Loo et al. 2012b). As these fetuses showed different patterns of anomalies, we assume that the phenotypic changes rather than underlying mutations caused the stellate cell anomaly. MLPA investigations in a renal agenesis fetus with abnormal WT1 expression in liver mesothelium suggested that *wt1* was not mutated in this fetus (Loo et al. 2012a). The *wt1*^−/−^ mouse model shows reduced liver size, abnormal stellate cell development and diaphragmatic defects (Ijpenberg et al. 2007). In these mice, Retinaldehyde dehydrogenase 2 (RALDH2) immunoperoxidase studies showed failure of mesenchymal cells to migrate along the body wall to the liver coelomic lining between E10.5 and E11.5, unlike control mice, because the pleuroperitoneal folds did not develop as in controls. In control mice, the liver was always in contact with the septum transversum and developing diaphragm and at E11.5–12.5, RALDH2 expression was prominent throughout the liver coelomic epithelium, but more weakly in the ventral area. In *wt1*^−/−^ mice RALDH2 staining was weaker than in control littermates and mainly restricted to the dorsal areas as most cells expressing RALDH2 accumulated at the site of the pleuroperitoneal defect, while fewer cells expressing RALDH2 reached the liver coelomic lining via other routes around adjacent organs (Ijpenberg et al. 2007). As RALDH2 is the main enzyme for production of retinoic acid in the fetus (Niederreither et al. 2003), this would result in reduced retinol production and presumably hepatic stellate cell expression of cRBP-1, as this is regulated by extracellular retinol levels (Jessen and Satre 2000). cRBP-1 immunohistochemistry shows almost no quiescent hepatic stellate cells in the fetus with complete diaphragm agenesis (Loo et al. 2015), reduced numbers in the fetuses with giant omphalocele and large diaphragmatic hernias and normal numbers of quiescent stellate cells in fetuses with smaller, isolated hernias. Many GFAP+ve mesenchymal cells were seen in these fetuses, suggesting a deficiency of cRBP-1 expression in stellate cells, probably reflective of reduced retinoic acid (Jessen and Satre 2000). The diaphragm develops around E8-11 in rodents, corresponding to weeks 4–6 in humans (Babiuk and Greer 2002). Previous reports suggested that liver mesenchymal cells derive from submesothelial cells around week 7 of gestation in humans (Loo and Wu 2008) or E12.5–E18.5 in mice (Asahina et al. 2009) and that the majority of stellate cells in this early stage of gestation are derived from mesothelium (Asahina et al. 2011).

Our findings also suggest there is another source of hepatic stellate cells. Stellate cells expressing GFAP antigen were seen in the fetus with bilateral diaphragm agenesis, where migration of mesodermal cells via the diaphragm (shown in animal experiments [Asahina et al. 2009]) would have been limited due to displacement of the liver into the thorax early in development and lack of development of the pleuroperitoneal membranes (Loo et al. 2015). This fetus showed abnormalities of the pancreatic head (which requires inductive signals from the septum transversum for normal development) but not of the pancreatic body (which normally receives inductive signals from mesenchyme around the notochord), suggesting either an abnormality of the septum transversum or loss of contact with septum transversum early in gestation, presumably due to herniation of pancreatic tissue into the thorax (Loo et al. 2015). The liver in this fetus would probably also have herniated into the thorax early in gestation, as the liver and pancreas are adjacent organs. A similar finding was reported in the *wt1*^−/−^ mouse model. Perivascular cells expressing desmin antigen (probably including stellate cells) are also present in normal numbers in the *wt1*^−/−^ mouse model where the pleuroperitoneal folds do not develop and immunoperoxidase stains showed fewer cells delaminating from the mesothelium (Ijpenberg et al. 2007). It has been proposed that hepatic stellate cells derive from bone marrow stromal cells, thought to be circulating MSC (Bianco 2011), although this finding is disputed (Yin et al. 2013). There are many similarities between stellate cells and MSC including ALCAM (activated leukocyte cell adhesion molecule) expression in stellate cell precursors (Asahina et al. 2009) and hemopoietic stromal cells (Ohneda et al. 2001), similar immunophenotype and osteoblastic, adipocytic, chondrogenic, and myoblastic differentiation potential (Castilho-Fernandes et al. 2011; Karaoz et al. 2011) and ability to support haemopoiesis (Kordes et al. 2013). Circulating stem cells in first-trimester human fetal liver express *α*SMA (Campagnoli et al. 2001). We found possible MSC in some fetal blood vessels that express GFAP, cRBP-1 and *α*SMA, which showed similar nuclear features to some hepatic stellate cells (Figs.1–5). Cells coexpressing GATA4 and RXR alpha but not desmin were noted in the liver in *wt1*^−/−^ mice, postulated to be progenitors of stellate or perivascular cells (Ijpenberg et al. 2007). Blast cells coexpressing vimentin and CD43 were seen in first and second trimester human fetal liver, which did not express hemopoietic markers (Timens and Kamps 1997). A subset of stellate cells expressing CD133, a progenitor cell marker, can be induced to form endothelial cells, stellate cells and hepatocyte-like cells in culture (Kordes et al. 2007). We previously reported that CD133 was expressed in some stellate cells mainly from the second trimester of gestation and later (Loo and Wu 2008). Hepatic stellate cells and haemopoiesis appear at similar times and appear to be linked in animal models (Oostendorp et al. 2002; Yin et al. 2013). C-X-C receptor type 4 (cxcr4) mediates homing of stellate cells and hemopoietic stromal cells (Son et al. 2006; Hong et al. 2009). In the diaphragm agenesis fetus we studied, ectopic liver nodules without mesothelium and ductal plates also lacked stellate cells and showed paucity of hemopoietic cells while both these cellular populations were present in other ectopic liver nodules with mesothelium and ductal plates, possibly because mesothelium and ductal plate cells express SDF-1 (Stromal-derived factor 1) (Coulomb-L’Hermin et al. 1999), the ligand for cxcr4. GFAP-positive cells were present in fetuses with reduced cRBP-1 stellate cells, including fetuses with diaphragm hernia and renal agenesis and GFAP labeled intravascular cells (Figs.2D and E, 5A), raising the possibility that this subset of hepatic stellate cells was derived from circulating cells. MSC can also travel via the vitelline veins (Collardeau-Frachon and Scoazec 2008) from the AGM to the liver. MesP1 lineage studies in mice show that MesP1-expressing progenitors contribute to hemopoietic stem cells and endothelial cells in the yolk sac and AGM regions (Chan et al. 2013). These endothelial cells are thought to be cells that support haemopoiesis (Ohneda et al. 2001). MesP1 is also expressed in embryonic and adult stem cells in mice and humans (Kordes et al. 2014) and in hepatic stellate cell precursors (Asahina et al. 2009). RALDH2 is necessary for haemopoiesis (Chanda et al. 2013) and is probably expressed in stromal cells supporting haemopoiesis such as in the AGM. Cells expressing RALDH2 were seen in developing diaphragm and liver coelom in control mice and were deficient in the *wt1*^−/−^ mice due to impaired migration caused by defects in the pleuroperitoneal folds (Ijpenberg et al. 2007). These findings raise the possibility that a common precursor is present for both cell types. It is possible that MSC circulate to the liver via both pathways but differentiate differently to produce a heterogenous stellate cell population with varying amounts of retinoic acid (D’Ambrosio et al. 2011). Our current study showed reduced delamination of cells from the mesothelium in renal agenesis fetuses with WT1 defects, while there appeared to be an increase in the numbers of circulating stem cells coexpressing *α*SMA and cRBP-1, suggesting that this may be an alternative pathway that may compensate for a lack of cells from the mesothelium, which may occur from the second trimester or later, unlike delamination of stellate cells from mesothelium which appears to occur earlier in development (Loo and Wu 2008). However, as we only used archival autopsy samples, it was not possible to examine all of the liver mesothelium and it is known that delamination of stromal cells from mesothelium is patchy (Loo and Wu 2008). Mesothelial cells can also give rise to hepatic stellate cells and myofibroblasts later in life, in experimental liver injury (Li et al. 2013). The finding of different immunophenotype in embryonic and later fetal hepatic stellate cells in animals (Kubota et al. 2007; Golbar et al. 2012) and human tissue (Geerts 2001; Loo and Wu 2008) also suggests that stellate cells change during development or different types of stellate cells are present in embryos and later in life, with different developmental origin – initially from mesothelium, then later from circulating stem cells. It is known that the pleuroperitoneal membranes do not develop in birds/chicks and that haemopoiesis does not occur in developing avian liver (Cumano and Godin 2007), while the septum transversum is present and stellate cells are known to delaminate from the coelomic lining in chicks (Perez-Pomares et al. 2004). However, the initial stages of avian liver development differ from those in mammals (Matsumoto et al. 2008), as does hemopoiesis in avian embryos, where the intermediate hemopoietic site occurs in the para-aortic region and not the liver (Cumano and Godin 2007). This is suggestive that hemopoietic stromal cells circulate to the liver via the diaphragm while hepatic stellate cells can reach the liver by different routes. Although these findings do not demonstrate conclusively that stellate cells are derived from circulating stem cells, they show that mesothelium is not the only source of stellate cells. There are other possible interpretations of our findings, for example, that the intravascular cells we describe are hepatocyte precursors, as these can express GFAP and *α*SMA in regenerating adult liver (Yang et al. 2008) and cRBP-1 is expressed in hepatocytes, although more weakly than in stellate cells (Figs. 1–3). There are also experimental studies showing that at least some hepatic stellate cells in adult mice are progenitor cells that can give rise to epithelial and mesenchymal cells in adult liver (Kordes et al. 2007, 2014; Yang et al. 2008) and lineage studies show that some of these cells are derived from precursors that express GFAP antigen (Yang et al. 2008). However, stellate cells from MesP1-expressing precursors do not give rise to hepatocytes or cholangiocytes after experimental liver injury (Lua et al. 2014), which would be consistent with the suggestion that there are different subpopulations of stellate cells (Wang et al. 2010). Alternative interpretations would require further experimental studies and are beyond the scope of our autopsy study. However, our observation of transitional forms between hepatic stellate cells and intravascular cells supports our interpretation that these are likely stellate cell precursors, in the contexts that we studied.

Other factors also influence hepatic stellate cell development. For example, increased stellate cell density was seen in fetuses with intrauterine growth restriction and ductal plate malformation (thought to be a ciliopathy), possibly due to abnormal Hedgehog signaling which is linked to primary cilia (Omenetti et al. 2011).

Our studies of renal agenesis fetuses with WT1 defects have shown diffuse increase in lobular *α*SMA expression (Loo et al. 2012b) (and in Fig.2), similar to the *wt1*^*−/−*^ mouse model (Ijpenberg et al. 2007) and beta catenin dermo-Cre mouse model (Berg et al. 2010). Stellate cells co-expressing *α*SMA and cRBP-1 were increased in a renal agenesis fetus with WT1 defects. WT1 is thought to regulate epithelial mesenchymal transition through the retinoic acid and beta catenin pathways, beta catenin deficiency causing precursor cells to differentiate into myofibroblastic rather than hepatic stellate cells (Berg et al. 2010). Our findings support the assertion that intact mesothelial function is required for normal stellate cell differentiation in human fetuses. Interestingly, human fetal liver stromal cells that support haemopoiesis also show high expression of regulators of Wnt signaling pathway (Martin and Bhatia 2005).

Our findings suggest that hepatic stellate cells may also derive from circulating MSC. We and others have observed fetal intravascular cells with features similar to hepatic stellate cells, expressing *α*SMA, GFAP and cRBP-1 antigens. Animal studies suggest that stellate cells migrate to the liver from the posterior mesoderm, via the diaphragm. We found hepatic stellate cells in displaced liver in fetuses with diaphragm agenesis and giant omphalocele, where the liver had limited contact with developing diaphragm and pleuroperitoneal membranes, suggesting an alternate source of stellate cells. Differentiation of hepatic stellate cells requires normal mesothelial function, as mesenchymal cells were abnormal in fetuses with WT1 defects in liver coelomic epithelium. Although we can only demonstrate these findings in small numbers of cases as the conditions are very rare, our observations complement animal studies and provide a means of translation of experimental data to human development.
